# Mélanocytose oculopalpébrale ou naevus d'Ota

**DOI:** 10.11604/pamj.2014.17.231.3540

**Published:** 2014-03-27

**Authors:** Hakima Elouarradi, Rajae Daoudi

**Affiliations:** 1 Université Mohammed V Souissi, Service d'Ophtalmologie A de l'hôpital des spécialités, Centre Hospitalier Universitaire, Rabat, Maroc

**Keywords:** Naevus d'Ota, naevus fusco-caeruleus, iris, evus of Ota, naevus fusco-caeruleus, iris

## Image en medicine

Le naevus d'Ota ou naevus fusco-caeruleus ophtalmo-maxillaris est une hyperpigmentation gris bleutée, voire brunâtre ou ardoisée unilatérale du visage dans le territoire cutané des 1ère et 2ème branches du nerf trijumeau. Il est plus fréquemment retrouvé chez les Asiatiques et les Noirs, rare chez les Européens. Les femmes sont près de cinq fois plus susceptibles d’être touchées que les hommes. Il est présent dès la naissance, mais il peut apparaitre plus tardivement dans la puberté. Il s'accompagne fréquemment d'une pigmentation oculaire touchant la sclérotique, la conjonctive, l'iris, la choroïde et la papille. Le glaucome chronique et La transformation en mélanome malin sont les risques principaux de cette pathologie justifiant une surveillance régulière. Les lésions cutanées peuvent être traitées sans séquelles cicatricielles par laser déclenché. Ce traitement nécessite en général de trois à une dizaine de séances. Nous rapportons le cas d'une patiente de 14 ans, sans antécédants particuliers présentant une mélanocytose oculopalpébrale congénitale. L'acuité visuelle est à 10/10. Le tonus oculaire est normal à 16 mmhg en ODG. L'examen du segment antérieur objective des lésions sclérales pigmentées sur 360°, un iris et un angle iridocornéen hyper pigmentés à la gonioscopie, et le fond d'oeil est normal. Un champ visuel est normal. Patiente est toujours suivie en consultation pour surveillance ophtalmologique régulière.

**Figure 1:**
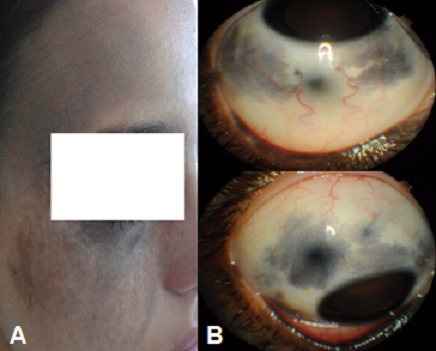
A) Mélanose oculopalpébrale; B) Iris hyperpigmenté, lésions pigmentées sclérales sur 360°

